# Synthesis and Characterization of Ti^4+^ Containing Carbonates Ti[CO_4_] and Ti_2_O_3_[CO_3_]

**DOI:** 10.1021/acs.inorgchem.5c03033

**Published:** 2025-10-27

**Authors:** Lkhamsuren Bayarjargal, Dominik Spahr, Victor Milman, Nico Giordano, Konstantin Glazyrin, Björn Winkler

**Affiliations:** † Institute of Geosciences, 9173 Goethe University Frankfurt, Altenhöferallee 1, Frankfurt 60438, Germany; ‡ Dassault Systémes BIOVIA, 334 Cambridge Science Park, Cambridge Cb4 0wn, United Kingdom; § 28332 Deutsches Elektronen-Synchrotron DESY, Notkestr. 85, Hamburg 22607, Germany

## Abstract

Two titanium carbonates, Ti­[CO_4_] and Ti_2_O_3_[CO_3_], were synthesized by the reaction of TiO_2_ with CO_2_ at high pressures and temperatures in a laser heated diamond anvil cell. Their structures were solved by in situ single crystal X-ray diffraction at high pressures. At pressures above ≈33 GPa, Ti­[CO_4_]-*I*4̅2*d* is stable. It transforms to Ti­[CO_4_]-I4_1_/*amd* on pressure release. The distinguishing feature of the Ti­[CO_4_]-structures is the presence of isolated [CO_4_]^4–^-groups, while Ti_2_O_3_[CO_3_] has isolated [CO_3_]^2–^-groups. Both structures contain 8-fold coordinated tetravalent Ti^4+^-cations. Full geometry optimizations based on DFT calculations reproduced the crystal structures. The DFT models were used to complement the experimental compression data and for the assignment of Raman modes. While carbonates are typically compressible compounds, Ti­[CO_4_] is rather incompressible with a bulk modulus of *K*_0_ ≈ 200 GPa. As we observed the pressure-induced transformation of TiO_2_, we provide the Raman spectra of TiO_2_–OI, and TiO_2_–OII.

## Graphical abstract

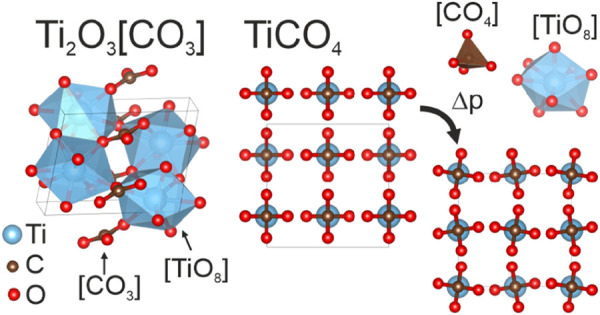

## Introduction

In the last 15 years, our understanding of the crystal chemistry of carbonates has profoundly changed. In earlier reviews of carbonates, the common and characteristic feature of the ≈280 “conventional” carbonates was the presence of trigonal [CO_3_]^2–^-groups (e.g. refs 1 and 2 ). High pressure research led to a significant extension of the field of carbonate crystal chemistry and crystallography. Specifically, single crystal diffraction studies at high pressures unambiguously showed, that at very high pressures the carbon atom prefers a tetragonal coordination, forming [CO_4_]^4–^-groups, and is *sp* ^3^-hybridized^3^ confirming the results of earlier powder diffraction based studies.^4^ Several *sp* ^3^-carbonates have been obtained since.^5–16,16–19^ In contrast to the conventional *sp* ^2^-carbonates, which have isolated [CO_3_]^2–^-groups, the [CO_4_]^4–^-groups can polymerize to form groups, rings, chains or pyramids. Their properties and stability fields are, however, still largely unexplored.

More recently, in situ high pressure studies of carbonates formed by reactions in laser heated diamond anvil cells have led to the discovery of pyrocarbonates, containing [C_2_O_5_]^2–^-groups.^20,21^ High pressure in situ single crystal studies have also shown that chemically simple carbonates, i.e., carbonates containing one type of metal cation only, with trivalent cations can be synthesized. Typical examples of the latter are carbonates containing trivalent cations such as Al_2_[CO_3_]_3_, Fe_2_[CO_3_]_3_, Cr_2_[CO_3_]_3_, and B­[μ-H­(CO_3_)_2_] which contain Al^3+^, Fe^3+^, Cr^3+^, and B^3+^ respectively.^22–25^ More recently, we synthesized an iodide carbonate ((IO_2_)_2_[CO_3_]) with a pentavalent iodide cation.^26^

The aim of the present study was to explore if a further extension of the crystal chemistry of carbonates is possible and if chemically simple anhydrous carbonates with tetravalent cations can be formed. Of course, a carbonate containing a Si^4+^ cation would be the most interesting phase, as it would be a potential storage material of carbon in the deep Earth. However, while some theoretical studies^27,28^ predicted structures containing [CO_3_]^2–^-groups in the SiO_2_–CO_2_ system, none of these predictions have been confirmed by experiments.^29–32^ Hence, we investigated the incorporation of Ti^4+^ instead. Ti^4+^ is the second most abundant tetravalent cation in the Earth’s mantle after Si^4+^, however, the abundance of titanium in the Earth is more than two orders smaller than that of silicon.^33^ Also, there is a significant difference between the ionic radius of Si^4+^ and Ti^4+^ in octahedral coordination (*r*(Si^4+^) = 0.4 Å *r*(Ti^4+^) = 0.6 Å), the preferred coordination at ambient conditions differs (Si^4+^ is typically found in tetrahedral coordination, whereas Ti^4+^ prefers octahedral coordination)^34,35^ and the electronic configuration is different. So while a structural analogy between SiO_2_ and TiO_2_ was suggested at megabar pressures^36^ structural correlations between Ti^4+^- and Si^4+^-bearing compounds may be limited at the moderate pressures investigated here.

CO_2_ and TiO_2_ have been found to coexist in inclusions of diamonds.^37^ This finding offers the possibility of the presence of titanium carbonates in the mantle at high pressure and temperature conditions, where the diamonds were grown. There are very few complex natural carbonate minerals, such as sabinaite (Na_4_Zr_2_TiO_4_[CO_3_]_4_), where tetravalent cations are incorporated by coupled substitution together with monovalent cations.^38^ Structures for hypothetical anhydrous Ti-carbonates, such as Ti­[CO_3_]_2_ and Ti_2_[CO_3_]_3_ with space group symmetry *Pa*3̅, have been predicted based on *ab initio* random structure searching technique.^39^ However, these predictions have not been experimentally validated and neither a natural simple titanium carbonate has been found nor has such a phase been synthesized experimentally up to now.

In summary, it is currently unclear whether anhydrous chemically simple carbonates with tetravalent cations can form at high pressure and temperature conditions and what kind of structure they will have. The aim of the present study therefore addresses the formation and characterization of chemically simple anhydrous carbonates with tetravalent Ti^4+^ cations. We present the successful synthesis, characterization by Raman spectroscopy and X-ray diffraction of two new Ti-carbonates with the composition Ti_2_O_3_[CO_3_] and Ti­[CO_4_] and discuss their unusual elastic properties.

## Methods

### High-Pressure Experiments

We used a synthetic TiO_2_ powder (99.5% purity, Alfa Aesar) and natural single rutile crystals (Großes Zirknitztal, Austria) for the high-pressure experiments. An additional characterization of the samples using SEM and energy dispersive X-ray spectroscopy (EDX) is provided in the Supporting Information (SI:EDX measurements of starting material and Figure S8).

The high-pressure experiments were carried out in diamond anvil cells (DACs) with Boehler-Almax design and 300 μm culet size.^40^ We used rhenium as gasket material which was preindented to a thickness of ∼45 μm. 120 μm gasket holes serving as sample chambers were drilled by a custom-built laser setup.

The compacted TiO_2_ powder or natural single crystals of rutile with maximum dimensions of up to ∼100 × 40 × 15 μm^3^ and a ruby for pressure determination were placed in the gasket hole. Then, CO_2_ was directly condensed into the sample chamber using a custom-built cryogenic loading system. In order to condense the CO_2_ into the sample chamber, a slightly opened DAC was placed on a liquid nitrogen cooled Cu-holder to cool down the DAC to ∼100 K. We used a small nozzle to direct a CO_2_ gas jet directly on the diamonds and the gasket.^21^ Ar was used as a purge-gas to avoid the precipitation of H_2_O ice. The precipitation of the CO_2_ in the gasket hole was monitored using an optical microscope. After a sufficient amount of CO_2_ was gathered in the sample chamber, the DAC was tightly closed. CO_2_ dry ice is less compressible than Ne or He, and hence the gasket hole does not shrink notably during compression.

The pressure was determined by measuring the shift of the ruby fluorescence assuming a potential error of 6% for deviations from hydrostatic conditions.^41^ We estimate a pressure uncertainty of at least ∼10% due to the pressure gradient and pressure change after laser heating. We additionally used the equation of states of the high pressure polymorphs of TiO_2_, i.e., of cotunnite (orthorhombic II (TiO_2_–OII)) and baddeleyite, as a pressure reference.^34,42,43^ This allowed us to independently constrain the pressure conditions in the X-ray diffraction experiments.

### Raman Spectroscopy and Laser Heating

High-pressure Raman spectroscopic measurements and the double-sided laser-heating were performed with a custom-built setup.^44^ Raman spectroscopy was carried out with a Nd:YAG laser (λ = 532.14 nm, Cobolt-Samba, Hübner Photonics) in combination with a Princeton Instruments ACTON SpectraPro (SP-2356) spectrometer equipped with a Pixis 256E CCD camera. We used a Raman laser spot size on the sample of ∼6 μm while using a laser power of 250 mW on the sample. Two-dimensional Raman maps were measured on a regular grid with a grid spacing of 6 μm. The Raman spectra were measured up to 2000 cm^–1^, depending on the pressure. The background correction was performed using the software package Matlab.

We used a pulsed CO_2_ laser (Coherent Diamond K-250) with λ = 10600 nm for double-sided laser-heating. The laser power was adjusted to 1–6 W to achieve coupling of the laser to the sample from both sides. Laser focusing on the sample results in a heating area of ∼40 × 40 μm^2^. The temperatures during the laser heating were determined by the two-color pyrometer method, employing Planck and Wien fits. Heating procedures in DACs may suffer from large temperature gradients, and the actual temperature is strongly dependent on the coupling of the laser with the sample. We estimate an uncertainty of at least ∼10% of the nominal temperature in the laser heated region depending on specific laser heating conditions, based on typical 2D temperature-gradient determination experiments performed in DACs.^45^

### Synchrotron Single Crystal Diffraction

Single crystal X-ray diffraction was performed at the PETRA III synchrotron (DESY) in Hamburg, Germany on the extreme conditions beamline P02.2.^46^ The beam size on the sample was ≈2 × 2 μm^2^ (fwhm), focused by Kirkpatrick-Baez mirrors. The diffraction data were collected using a PerkinElmer XRD1621 detector using either the wavelength of 0.2901 Å or 0.2904 Å. We collected multigrain single crystal data while rotating by ±33° around the axis perpendicular to the beam in the DAC while collecting frames in 0.5° steps with 4 s acquisition time per frame.

The detector to sample distances (412 mm and 425 mm) were calibrated with a CeO_2_ standard using DIOPTAS.^47^ At each experimental session, the instrument calibration was done using data collected from a single crystal of enstatite (MgSiO_3_). After the measurement, the reflections were indexed and integrated employing CrysAlis^PRO^. We used the Domain Auto Finder program (DAFi) to find possible single crystal domains for the subsequent data reduction.^48^ The crystal structures in this study were solved using SHELXT.^49,50^ After structure solution, the crystal structures were refined with SHELXL.^51^ SHELXT and SHELXL programs were implemented in the Olex2 software package.^52^ Some structure refinements were performed using the software package JANA2006.^53^

### Density Functional Theory-Based Calculations

First-principles calculations were carried out within the framework of density functional theory (DFT), employing the Perdew–Burke–Ernzerhof (PBE) exchange-correlation functional and the plane wave/pseudopotential approach implemented in the CASTEP simulation package.^54–56^ “On the fly” norm-conserving or ultrasoft pseudopotentials generated using the descriptors in the CASTEP database were employed in conjunction with plane waves up to a kinetic energy cutoff of 1020 or 630 eV, for norm-conserving and ultrasoft pseudopotentials, respectively. The accuracy of the pseudopotentials is well established.^57^ A Monkhorst–Pack grid was used for Brillouin zone integrations.^58^ We used a distance between grid points of <0.023 Å^–1^. Convergence criteria for geometry optimization included an energy change of <5 × 10^–6^ eV atom^–1^ between steps, a maximal force of <0.008 eV Å^–1^ and a maximal component of the stress tensor <0.02 GPa.

Elastic stiffness coefficients were obtained by the strain–stress method. In the stress–strain method employed here, symmetry adapted-strain patterns were imposed on the fully optimized ground state structure. For each symmetry adapted strain atomic coordinates were relaxed, and the stress tensor was obtained for three to six different amplitudes. Elastic coefficients and their statistical errors were obtained from linear fitting of the stress–strain dependencies.^59^

Phonon frequencies were obtained from density functional perturbation theory (DFPT) calculations.^60,61^ Raman intensities were computed using DFPT with the “2*n* + 1” theorem approach.^62^

## Results and Discussion

### Compression and Heating of TiO_2_ + CO_2_

Raman spectra of the mixture TiO_2_ + CO_2_ were obtained after laser heating up to 2600(300) K at 25, 35, and 40 GPa. During heating, CO_2_–III transformed into CO_2_–IV and partially into CO_2_–V. These transitions can be conveniently followed by Raman spectroscopy. A sequence of high pressure polymorphs of TiO_2_ are expected when rutile is used as precursor.^34,42,43^ We obtained TiO_2_–OI at around 20 GPa, which eventually transformed to TiO_2_–OII at above 38 GPa. No carbonates were formed after heating at pressures 25 and 35 GPa, but a reaction occurred once we laser heated for a prolonged time at ∼40 GPa (Figure 1).

**Figure 1:**
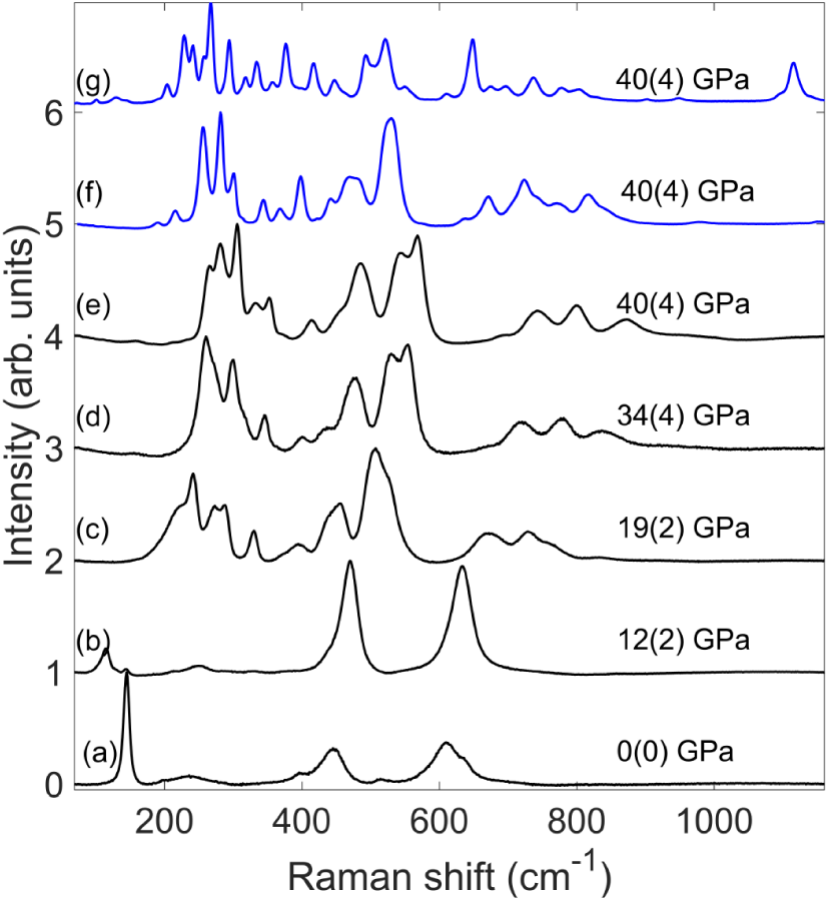
(a) Raman spectrum of starting sample TiO_2_-rutile at ambient conditions. (b–d) Raman spectra of TiO_2_ during compression without heating. (e) Raman spectra of samples before laser heating at 40(4) GPa. (f) Raman spectra of samples after short laser heating at 40(4) GPa, with conbtributions by TiO_2_–OII and TiO_2_–OI. (g) Raman spectra of samples after long laser heating at 40(4) GPa. The appearance of a Raman band close to 1200 cm^–1^ is indicative of the presence of a carbonate. There are Raman bands due to the new carbonate, TiO_2_–OII and CO_2_–V.

### Structure of TiO_2_–OII

From the single crystal X-ray diffraction experiment, we determined the structure of the high pressure polymorph of TiO_2_–OII. It corresponds to the structure which was found in previous studies.^63,64^ At 42 GPa, the lattice parameters of TiO_2_–OII are *a* = 5.1097(7) Å, *b* = 2.9612(13) Å, *c* = 5.944(4) Å and *V* = 89.94(7)­Å^3^. (*Pnma*, *Z* = 4). The Ti atoms are 9-fold coordinated by oxygen with Ti–O-distances ranging from 1.932(4) to 2.259(4) Å with an average distance of 2.09(13) Å. The pressure-dependence of the unit-cell volume of TiO_2_–OII phase is shown together with previous data of TiO_2_–OII in Figure S1.^42^ Tables S1 and S2 show our crystallographic data of TiO_2_–OII, which has been deposited in the Cambridge Structural Database (CSD) under deposition numbers CCDC 2485339.

### Structure of Ti_2_O_3_[CO_3_]-*P*2/*c*

When heating TiO_2_–OII, in three distinct experiments we observed new additional Raman peaks after laser heating above 40 GPa. This indicated the reaction of TiO_2_ with the surrounding CO_2_ and the formation of two new phases. The analysis of the synchrotron single crystal X-ray diffraction data of the first unknown phase showed that this was a titanium carbonate with the composition Ti_2_O_3_[CO_3_]. After the data reduction the crystal structure was solved in the monoclinic space group *P*2/*c* (No. 13) with *Z* = 2. The lattice parameters at 39(3) GPa are *a* = 6.4859(10) Å, *b* = 4.213(3) Å, *c* = 4.990(2) Å β = 101.71(2)° and *V* = 133.51(11) Å^3^ (Tables S3–S6).

The structural parameters derived from experiments were employed as a trial structure in DFT calculations. The structure after the full geometry optimization was in good agreement with the experimental values, as the DFT calculations gave *a* = 6.4614 Å, *b* = 4.3257 Å, *c* = 5.0041 Å β = 101.785° and *V* = 136.92 Å^3^ (Table S3).

This crystal structure contains isolated [CO_3_]^2–^-groups and Ti^4+^ cations. The Ti atoms are 8-fold coordinated by oxygen with Ti–O-distances ranging from 1.804(6) to 2.282(4) Å. The average Ti–O-distance is 2.02(16) Å. These interatomic distances are well in the range of typical Ti–O-distances in TiO_2_–OII, which were determined by us at 42 GPa. The coordination polyhedra of the Ti-atoms form a network by sharing edges and corners. All carbon atoms are coordinated by three oxygen atoms forming a distorted triangle. The [CO_3_]^2–^-groups of Ti_2_O_3_[CO_3_] have one short (∼1.213(12) Å) and two longer (∼1.270(6) Å) C–O bonds. The shortest C–O bond is connected with the longest Ti–O bonds in the coordination polyhedra of Ti. Also, one O–C–O angle $\phi_{1}^{O-C-O}$ =115.3­(7)° differs significantly from the other two angles with $\phi_{2,3}^{O-C-O}$ =122.4­(4)° in [CO_3_]-groups. The coordination [TiO_8_]-polyhedra and [CO_3_]-groups are illustrated in Figure 2a. Each “double layers” of [TiO_8_]-polyhedra are connected to each other with [CO_3_]-groups (Figure 2a). The crystallographic data of Ti_2_O_3_[CO_3_]-*P*2/*c* has been deposited in the Cambridge Structural Database (CSD) under the deposition number CCDC 2416531.

**Figure 2:**
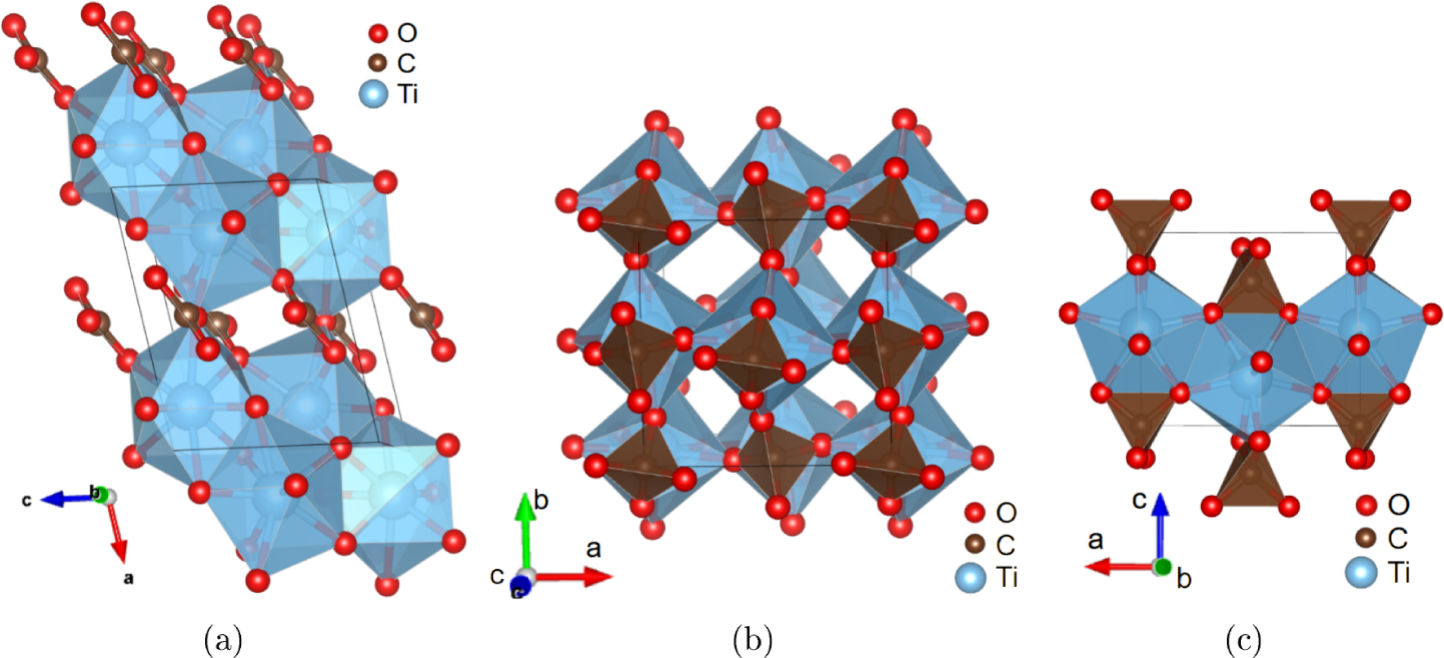
(a) The structure of Ti_2_O_3_[CO_3_] at 39(3) GPa, viewed along [010]. The structure contains isolated [CO_3_]-groups and [TiO_8_]-polyhedra. Each double layers of [TiO_8_]-polyhedra are connected to each other with CO_3_-groups. (b) The structure of Ti­[CO_4_] at 39(3) GPa, viewed along [001]. The [TiO_8_]-polyhedra and CO_3_-groups are main building blocks of Ti­[CO_4_]. (c) The structure of Ti­[CO_4_] at 39(3) GPa, viewed along [010].

### Structure of Ti­[CO_4_]-*I*4̅2*d*

The crystal structure solution and refinement of the second new phase showed that this is a novel inorganic titanium carbonate with Ti­[CO_4_] composition. At 39(3) GPa, Ti­[CO_4_] crystallizes in the tetragonal space group *I*4̅2*d* with *Z* = 4, a = 5.8124(9) Å, *c* = 5.080(3) Å and *V* = 171.62(11)­Å^3^. The structure of Ti­[CO_4_]-*I*4̅2*d* is shown in Figure 2b. This structure contains isolated [CO_4_]^4–^-carbonate groups and Ti^4+^. The Ti^4+^ atom is 8-fold coordinated by oxygen with two different Ti–O-distances 1.9241(19) Å and 1.979(3) Å. The average bond Ti–O length is 1.9515 Å and is thus slightly shorter than Ti–O-distances in TiO_2_–OII or Ti_2_O_3_[CO_3_]. The [TiO_8_] polyhedra are irregularly shaped and form a network by sharing edges of polyhedra. Each­[CO_4_]-tetrahedra shares two edges with two different [TiO_8_]-polyhedra.

The crystallographic parameters and details of the structure refinement of Ti­[CO_4_]-*I*4̅2*d* measured at 39(3) GPa are listed in Tables S7 and S8. The crystallographic data of Ti­[CO_4_]-*I*4̅2*d* has been deposited in the Cambridge Structural Database (CSD) under the deposition number CCDC 2416536.

The structural model derived from the experiments was employed as a trial structure for DFT full geometry optimizations. The two data sets are in the expected good agreement. A comparison can be found in Table S7.

### Structure of Ti­[CO_4_]-*I*4_1_/*amd*

On pressure release at lower pressures, Ti­[CO_4_]-*I*4̅2*d* undergoes a phase transition to a structure with the higher symmetry *I*4_1_/*amd*. At 14.4(30) GPa, the crystal structure was solved and it was found that Ti­[CO_4_] crystallizes in the tetragonal space group *I*4_1_/*amd* with *Z* = 4 (Figure 3).

**Figure 3:**
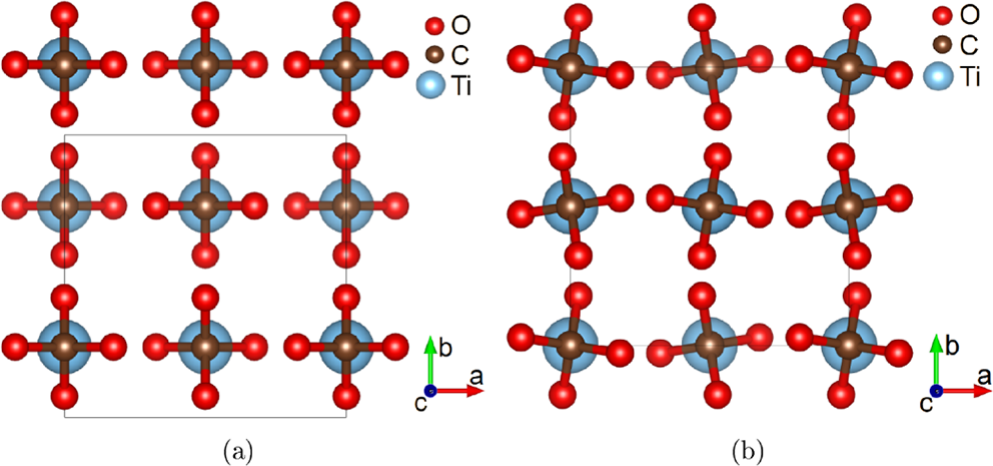
Structure viewed along [001] for (a) the low-pressure phase Ti­[CO_4_]-*I*4_1_/*amd* at 14.4(30) GPa and (b) the high pressure phase Ti­[CO_4_]-*I*4̅2*d* *I*4̅2*d* at 39(3) GPa. The displacement of the oxygen atoms is responsible for the phase transition between both structures.

The structure solution results in unit cell parameters *a* = *b* = 6.021(4) Å and *c* = 5.185(2) Å for Ti­[CO_4_]-*I*4_1_/*amd*. In Ti­[CO_4_]-*I*4_1_/*amd* there is one symmetrically independent Ti–O distance of 2.003(4) Å between Ti and O atoms, while in Ti­[CO_4_]-*I*4̅2*d* there are two symmetrically independent Ti–O-distances. All carbon atoms are tetrahedrally coordinated by oxygen atoms, where the interatomic Ti–O distances are 1.355(3) Å at 39(3) GPa and 1.374(3) Å at 14.4(30) GPa.

The Ti­[CO_4_]-*I*4_1_/*amd* structure is very similar to the structure of Ti­[CO_4_]-*I*4̅*d* at 39(3) GPa. The main difference is due to a displacement of the oxygen atoms from a special position in *I*4_1_/*amd* to the general position in *I*4̅2*d* with a concomitant rotation of the [CO_4_] tetrahedra around the *c*-axis. The displacement of the oxygen atoms is clearly discernible from a comparison of crystal structures at pressures of 39(3) GPa and of 14.4(30) GPa in Figure 3.

The crystallographic parameters and details of the structure refinement of Ti­[CO_4_]-*I*4_1_/*amd* measured at 14.4(30) GPa are listed in Tables S7 and S9. The crystallographic data of Ti­[CO_4_]-*I*4_1_/*amd* has been deposited in CSD under the deposition number CCDC 2416537. Here again, the structural parameters obtained from DFT full geometry optimizations are in good agreement with those determined experimentally.

### Phase Transition of Ti­[CO_4_]

Our DFT calculations predict that the phase transition occurs around 31–35 GPa. In this pressure range, the volume of Ti­[CO_4_]-*I*4̅2*d* is ≈0.7% larger than that of Ti­[CO_4_]-*I*4_1_/*amd* (Figures 4 and S5). At ≈30 GPa, X-ray diffraction data shows still Ti­[CO_4_]-*I*4̅2*d*. Unfortunately, no X-ray data were collected between 30 and 14.4 GPa and therefore the accuracy of the predicted transition pressure cannot currently be assessed.

**Figure 4:**
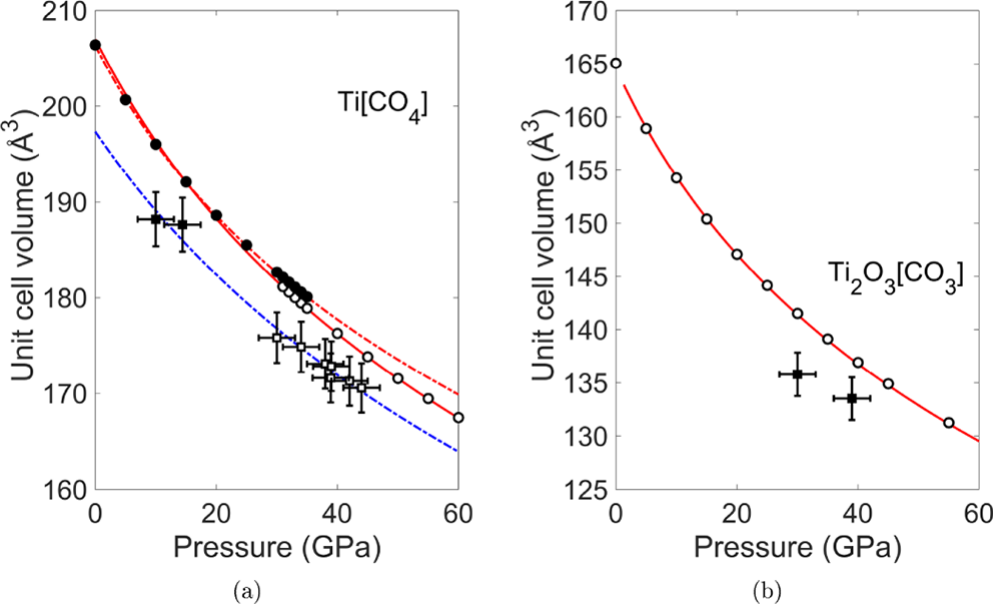
(a) Pressure-dependence of the unit-cell volumes of Ti­[CO_4_]. The dashed lines represents BM3-EOS which were fitted to the DFT data of Ti­[CO_4_]- $I4_{1}/amd$ (black circles) and Ti­[CO_4_]-*I*4̅2*d* (open cycles). The dashed blue line marks BM3-EOS fit of experimental data of Ti­[CO_4_] (squares). (b) Pressure-dependence of the unit-cell volumes of Ti_2_O_3_[CO_3_]. The DFT data (circles) were fitted using BM3-EOS (red line). Open squares show the experimental data.

The crystal structures and chemical compositions of Ti_2_O_3_[CO_3_] and Ti­[CO_4_] differ significantly from the previously predicted Ti-carbonate phases such as Ti­[CO_3_]_2_ and Ti_2_[CO_3_]_3_ with space group symmetry *P*a3̅.^39^ Instead, Ti­[CO_4_]-*I*4_1_/*amd* is isostructural to zircon, Zr­[SiO_4_]. In Zr­[SiO_4_] a phase transition from *I*4_1_/*amd* into *I*4̅2*d* was first predicted by calculations^65^ and later confirmed by experiment.^66^ This phase transition occurs 21.0(1) GPa and is driven by a Raman active soft-mode. In Ti­[CO_4_]-*I*4_1_/*amd*, a *A*_*u*_ mode becomes soft at the Γ-point with increasing pressure, but this mode is not Raman active.

### Bulk Modulus of Ti_2_O_3_[CO_3_] and Ti­[CO_4_]

The bulk modulus of both phases was obtained by fitting a third-order Birch–Murnaghan equation (BM3-EOS)^67,68^ of states to volumes obtained by experiments and DFT-based calculations (Figure 4) using the EoSFit7 program.^69^ The bulk moduli from BM3-EOS are given in Table 1 .

**Table 1:** Bulk Modulus Were Determined Using the Third-Order Birch-Murnaghan equation^67,68^ of States (BM3-EOS) from Theoretical and Experimental Volumes of Unit Cells and Coordination Polyhedra and Using Stress-Strain Calculations (See Figure 4)

	Third-order BM-EOS		
0–60 GPa	*K*_0_ (GPa)	*V*_0_ (Å^3^)	*K*_*p*_
Ti_2_O_3_[CO_3_] (calc.)	131(2)	164(1)	5.4(1)
[TiO_8_] in Ti_2_O_3_[CO_3_] (calc.)	139(3)	17.45(2)	5.5(1)
Ti[CO_4_] (exp.)	212(25)	197(2)	4.5(1.8)
Ti[CO_4_] (calc.)	192(9)	206(1)	3.9(3)
Ti[CO_4_]-*I*4_1_/amd (0–35 GPa, calc.)	166(1)	206.34(4)	6.5(1)
Ti[CO_4_]-*I*4̅2*d* (31–65 GPa, calc.)	166(1)	207.00(4)	5.0(1)
[TiO_8_] in Ti[CO_4_] (calc.)	148(8)	16.07(4)	8.0(5)
[CO_4_] in Ti[CO_4_] (calc.)	397(24)	1.37(2)	8.9(1.2)
[CO_4_] in Ti[CO_4_] (0–35 GPa, calc.)	435(2)	1.3713(1)	4.1(1)
[CO_4_] in Ti[CO_4_] (31–65 GPa, calc.)	468(2)	1.3676(3)	5.9

The theoretical bulk modulus of Ti_2_O_3_[CO_3_] obtained from BM3-EOS is *K*_0_ = 131(2) GPa for the calculated data between 0 and 60 GPa. This value is similar to highest bulk moduli (131 and 125 GPa) of carbonates with isolated [CO_3_]-groups such as Ni­[CO_3_] or Co­[CO_3_] at ambient conditions.^70,71^

The complete elastic stiffness tensor was obtained for the ambient pressure structures of Ti­[CO_4_]-*I*4̅2*d* and Ti­[CO_4_]-*I*4_1_/*amd* from stress–strain calculations (Table S10). The bulk moduli computed from the elastic stiffness tensor are *K*_0_(Ti­[CO_4_]-*I*4_1_/*amd*) = 156(2) GPa and *K*_0_(Ti­[CO_4_]-I4̅2d) = 157(2) GPa.

Considering the bulk moduli obtained from BM3-EOS fits to theoretical data, both phases have same the bulk modulus *K*_0_ = 166(1) GPa (Table 1 ), which is smaller than bulk moduli *K*_0_ = 201(3) GPa of Ti­[CO_4_]-*I*4_1_/*amd* and *K*_0_ = 204(1) GPa of Ti­[CO_4_]-*I*4̅2*d* from a fit with a BM2-EOS.

As expected, the bulk modulus of Ti­[CO_4_] is much higher than the bulk modulus of Ti_2_O_3_[CO_3_] or carbonates with isolated [CO_4_]-groups (Ca_2_CO_4_: *K*_0_ = 108(10) GPa.^16^ It is smaller than the bulk modulus (*K*_0_ = 227 GPa) of the isostructural silicate zircon.^72^ Based on previously published bulk moduli of carbonates,^16,19–70,71,73,74^ Ti­[CO_4_] is highly likely the most incompressible compound within the broad carbonate family.

### Compression of Ti_2_O_3_[CO_3_] and Ti­[CO_4_]

To understand the origin of the relatively high bulk modulus of Ti­[CO_4_], we analyzed the compressibility of the coordination polyhedra and tilting angles in both structures. Figure 5 shows the DFT-calculated volumes of the CO_4_ and [TiO_8_] polyhedra together with fits using a BM3-EOS.

**Figure 5:**
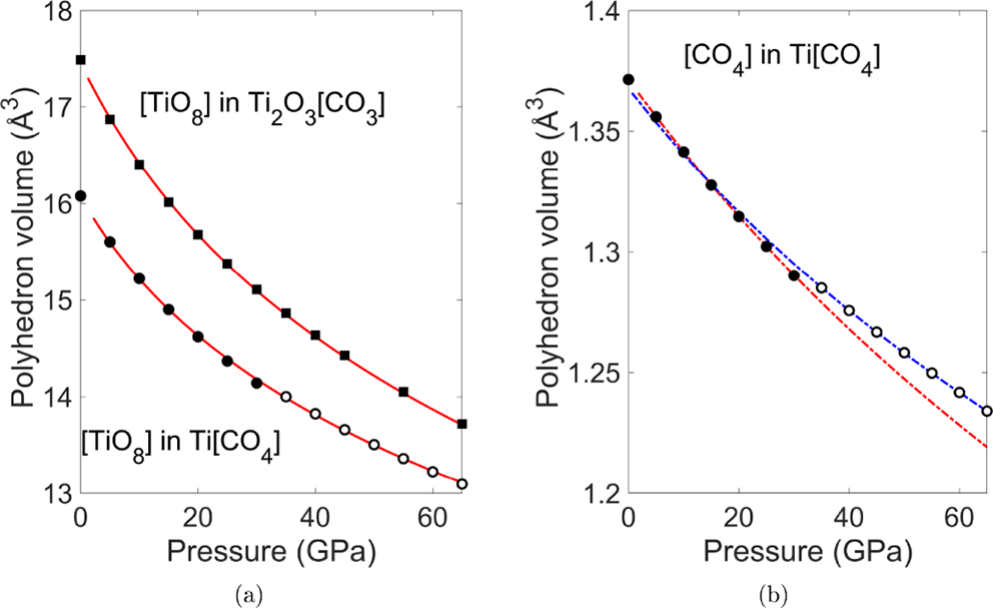
(a) Pressure-dependence of volumes of [TiO_8_] polyhedra. The [TiO_8_] polyhedron volumes of Ti­[CO_4_] are represented by open symbols and the corresponding polyhedron volumes of [TiO_8_] in Ti_2_O_3_[CO_3_] by black symbols. The DFT data (circles) were fitted using and BM3-EOS (red lines). (b) Pressure-dependence of volumes of [CO_4_]-polyhedra in Ti­[CO_4_]. The dashed blue line and open cycles represent BM3-EOS fit and DFT data of Ti­[CO_4_]-*I*4̅2*d*. The dashed red line and black cycles represent BM3-EOS fit and DFT data of Ti­[CO_4_]-*I*4_1_/*amd*.

The bulk modulus of the [TiO_8_] polyhedra in Ti_2_O_3_[CO_3_] is 139(3) GPa. The compressibility of Ti_2_O_3_[CO_3_] is anisotropic (Figure 6a), where the *c*-axis is significantly less compressible than the *a*- and *b*-axes. This can be rationalized by noting that the [CO_3_]^2–^-groups are essentially rigid and the [TiO_8_] polyhedra are rather incompressible so that the major pressure induced structural changes are changes C–O–Ti angles. This is shown in Figure 7b,d, where the C–O–Ti angles ω_3_ and ω_4_ are strongly pressure-dependent, while the Ti–O–Ti angle ρ_2_ increases only slightly. The effect of this is that the distance between the “double layers” shown in Figure 2a containing the [TiO_8_]-polyhedra decreases on pressure increase, which makes the *a*-axis compressible. As there is a tilt between the [CO_3_]-groups and the [TiO_8_]-polyhedra, the *b*-axis is also compressible. The *c*-axis is rather incompressible, as a shortening of this axis would imply a decrease in Ti–Ti distances in the edge-sharing [TiO_8_]-polyhedra, which is energetically unfavorable.

**Figure 6:**
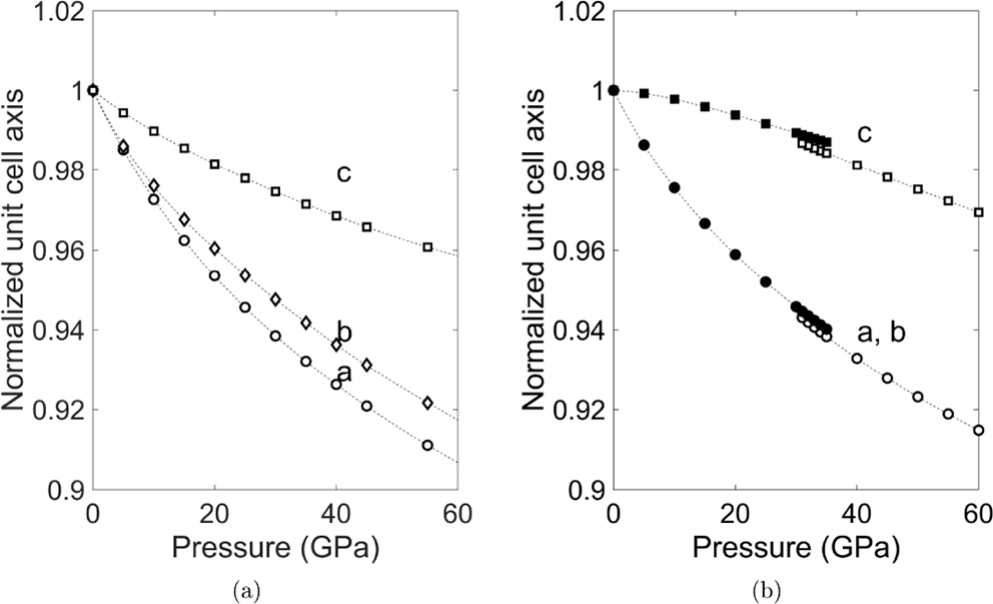
(a) Pressure dependence of the relative unit-cell parameters of Ti_2_O_3_[CO_3_] (b) Pressure dependence of the relative unit-cell parameters of Ti­[CO_4_] Filled symbols represent the Ti­[CO_4_]-*I*4_1_/*amd* phase, while open symbols refer to the Ti­[CO_4_]-*I*4̅2*d* phase. Dashed lines are guides to the eye.

**Figure 7:**
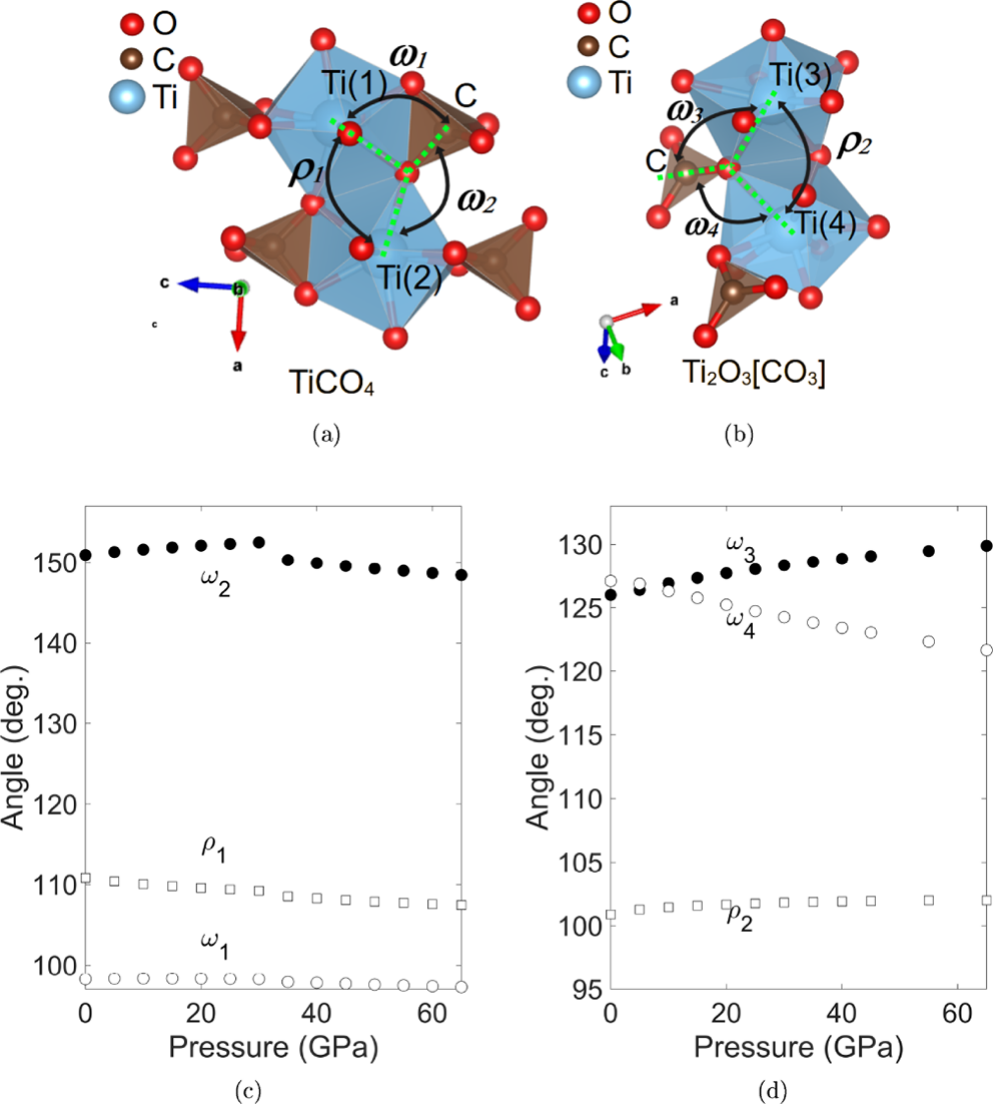
(a, b) Pressure-dependence of the angles between different atoms in Ti­[CO_4_] and Ti_2_O_3_[CO_3_]. (c) Filled circles represent the ω_2_ angle between C–O–Ti(2) in Ti­[CO_4_]. Open circles represent the ω_1_ angle between C–O–T(1) and squares the ρ angle between Ti(1)–O–Ti(2) in Ti­[CO_4_]. (d) Filled circles represent the ω_3_ angle between C–O–Ti(3) in Ti_2_O_3_[CO_3_]. Open circles represent the ω_4_ angle between C–O–Ti(4) and squares the angle between Ti(3)–O–Ti(4) in Ti_2_O_3_[CO_3_].

The bulk modulus of the TiO_8_ polyhedra in Ti­[CO_4_] is 148(7) GPa, which is slightly higher than the value for the related polyhedra in Ti_2_O_3_[CO_3_]. This is consistent with the slightly smaller volume of the [TiO_8_] polyhedra in Ti­[CO_4_] (V_0_(Ti­[CO_4_]) = 15.85(6) Å^3^) compared to that in Ti_2_O_3_[CO_3_] (V_0_(Ti_2_O_3_[CO_3_]) = 17.30(5) Å^3^) (Figure 5a).

The [CO_4_]-groups in Ti­[CO_4_] have a large bulk modulus, *K*_0_ = 397 (24) GPa, due to the strong covalent bonding between the C- and O-atoms (Figure 5b and Table 1). Within the substantial error this value is similar to the bulk moduli of [CO_4_]-groups in other carbonates such as Ca_2_CO_4_ (360(38) GPa).^16^ It is slightly larger than the polyhedral bulk modulus of [SiO_4_]-groups in most silicate garnets.^75^

The bulk modulus of Ti­[CO_4_] is in between the bulk moduli of the [TiO_8_] polyhedra and [CO_4_]-groups. The axial compression behavior of the *c*-axis is unusual in that it is slightly concave at low pressures for Ti­[CO_4_]-*I*4_1_/*amd*. These two observations can be correlated again with the topology and the pressure-induced rotation of the constituent polyhedra. The angles are shown in Figure 7a, where ω_1_ and ω_2_ are C–O–Ti angles, while ρ_1_ is a Ti–O–Ti angle. As is shown in Figure 7c, ω_2_ increases during the compression of the Ti­[CO_4_]-*I*4_1_/*amd*-phase, then discontinuously jumps to a lower value at the phase transition, and decreases on further compression. All other angles remain essentially unchanged. The consequence of this change is that essentially rigid units comprising a [TiO_8_] and two [CO_4_]-groups (Figure 7a) move with respect to one another, leading to compressible *a*- and *b*-axes and the concave behavior of *c*-axes in Ti­[CO_4_].

### Raman Spectroscopy

Spatially resolved Raman measurements allowed us to locate the individual phases in the DAC after laser heating. This is shown in Figure 8.

**Figure 8:**
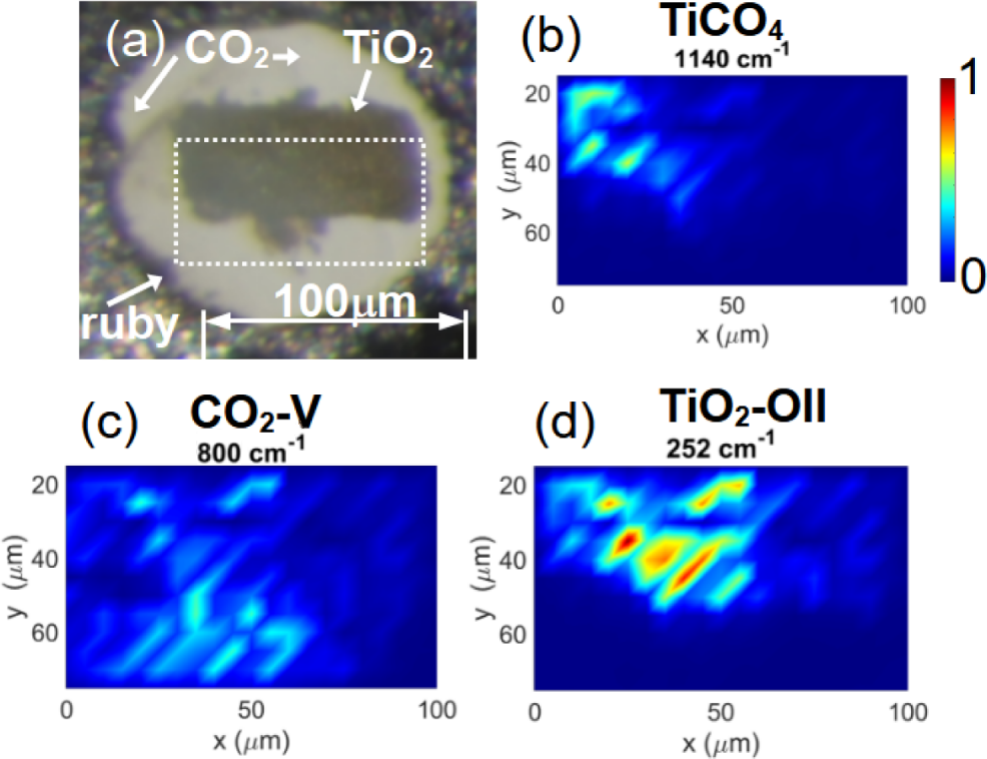
(a) Optical microscopic image of compacted TiO_2_ powder and CO_2_ in the DAC before laser heating at 40(4) GPa. The area in which spatially resolved Raman spectra were collected is indicated by a dashed square. After laser heating and temperature quenching, three phases could be identified at 40 GPa. (b) A Raman map of the 1140 cm^–1^ band shows the location of Ti­[CO_4_], (c) the distribution of CO_2_–V was mapped by the band at 800 cm^–1^ (d) TiO_2_–OII was located by mapping a Raman band at 252 cm^–1^.

DFPT-calculations of the Raman spectra complemented the measurements and provided a link between structural and spectroscopic properties. The Raman map allowed us to select regions in which mainly a single phase, either TiO_2_–OI, TiO_2_–OII, CO_2_–V or Ti­[CO_4_]-*I*4̅2*d* could be measured. In Figure 9 experimental and DFPT-Raman spectra obtained at different locations in the DAC are compared to each other.

**Figure 9:**
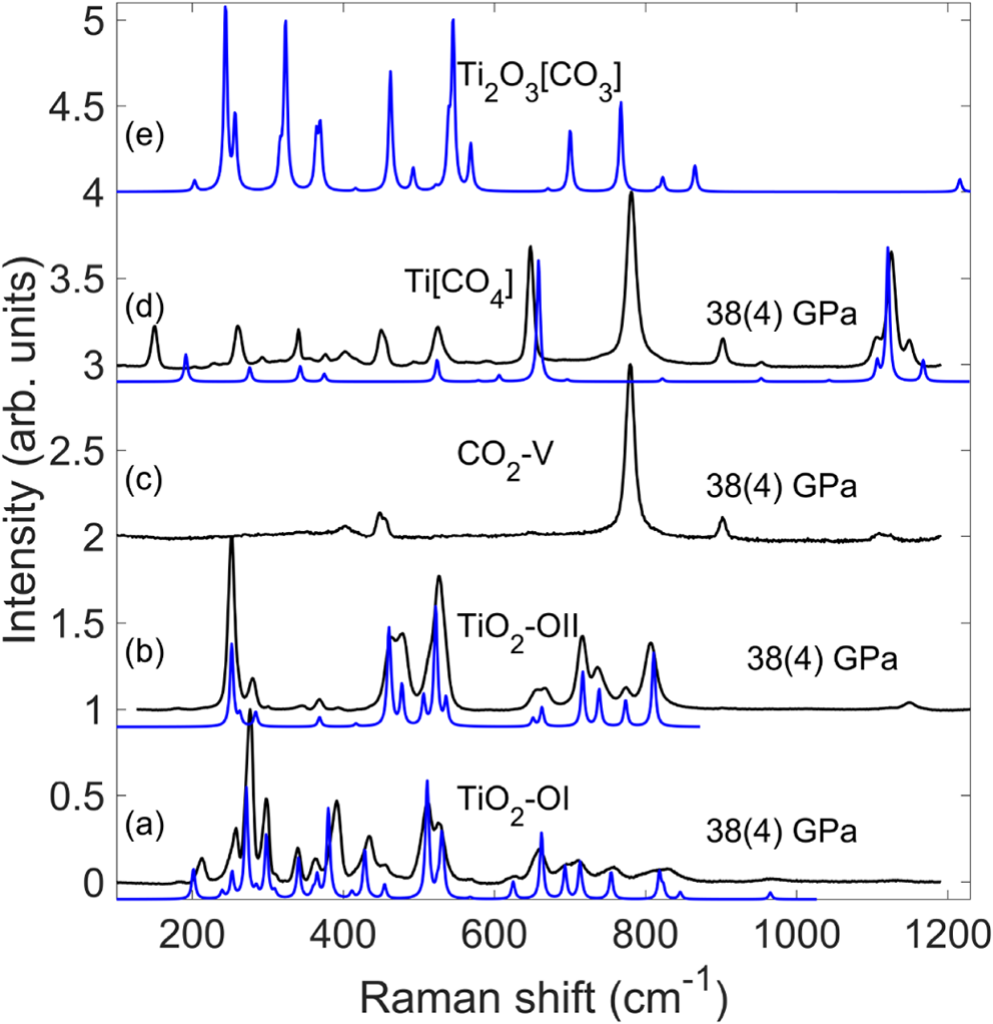
Raman spectra of TiO_2_–OI (a), TiO_2_–OII (b), CO_2_–V (c), Ti­[CO_4_]-*I*4̅2*d* (d) and Ti_2_O_3_[CO_3_] (e). Experimental Raman spectra (black line) were collected at 38(4) GPa after laser heating quenched to ambient temperature. Theoretical (blue line) Raman spectra frequencies were calculated at 40 GPa and scaled by 4.1% to account for the well-established GGA-PBE underbinding.

As can be seen from Figure 9a, the most intense Raman band of TiO_2_–OI can be found at ∼277 cm^–1^ at pressure of 38(4) GPa. Three further intense bands are located at ∼298, 391, and 511 cm^–1^. To the best of our knowledge, this is the first time a Raman spectrum of TiO_2_–OI has been obtained. The DFPT model calculations reproduce the spectrum very well, thus allowing an assignment of bands to eigenvectors.

Similarly, at 38(4) GPa, the most intense Raman band of TiO_2_–OII is located at ∼252 cm^–1^ and another prominent Raman band is at ∼526 cm^–1^ (Figure 9b). More than 11 other modes of TiO_2_–OII were observed in a range of 250–807 cm^–1^. This is also the first time a Raman spectrum of TiO_2_–OII has been published. Here again, the DFPT calculations reproduce the experimental spectrum well and can be employed to understand the corresponding displacement vectors.

After the reaction was induced by laser heating, characteristic Raman modes of Ti­[CO_4_] can be observed unequivocally at ∼648 and ∼1125 cm^–1^, in addition to the Raman modes of TiO_2_ and CO_2_–V Figure 9). The irreducible representations of Ti­[CO_4_]-*I*4̅2d at the Γ-point result in Γ_tot_ = 3A_1_ + 3A_2_ + 5B_1_ + 5B_2_ + 10E. The acoustic phonons are Γ_acoustic_ = B_2_ + E. The Raman active modes are Γ_Raman_ = 3A_1_ + 5A_2_ + 4B_1_ + 9E while the Γ_IR_ = 4B_2_ + 9E modes are infrared active. We have clearly identified 11 of the 21 Raman active modes at ambient conditions in the range between 60 cm^–1^ and 1200 cm^–1^ at 38(4) GPa. Using the DFPT calculations, which reproduce the Raman spectrum very well, we found that the characteristic band at 1125^–1^ with very strong intensity can be attributed to the vibration of Ti-Atoms along the *c*-axis (Figure 9d). The second intense Raman band of Ti­[CO_4_] is separated from other bands at ∼648 cm^–1^, and reflects the vibration of oxygen atoms in the­[CO_4_]-groups. Lattice modes with similarly intense peaks have energies of 525, 341, 260, and 151 cm^–1^.

In Ti_2_O_3_[CO_3_]–*P*2/*c*, each unit cell contains two formula units and hence there are 54 normal modes (Γ_tot_ = 12A_*g*_ + 12A_*u*_ + 15B_*g*_ + 15B_*u*_). After subtraction of the 3 acoustic phonons (Γ_acoustic_ = A_*u*_ + 2B_*u*_), the irreducible representations of the optical vibrations of Ti_2_O_3_[CO_3_] at the Γ-point is Γ_optic_ = 12A_*g*_ + 11A_*u*_ + 15B_*g*_ + 15B_*u*_ where Γ_Raman_ = 12A_*g*_ + 15B_*g*_ are Raman active while the infrared active modes have representations Γ_IR_ = 11A_*u*_ + 13B_*u*_. Our calculated Raman spectrum of Ti_2_O_3_[CO_3_] shows five strong characteristic Raman modes between 240–550 cm^–1^ and a very weak symmetric stretching mode of [CO_3_]-groups around 1216 cm^–1^ (Figure 9). Unfortunately, these strong peaks overlapped with the Raman peaks of the major phases such as Ti­[CO_4_] and TiO_2_ in our experiments. Because Ti_2_O_3_[CO_3_] was present in small quantities as a minor phase only, it was impossible to collect a clear Raman spectrum of this phase.

### Stability

We have shown that Ti_2_O_3_[CO_3_] and Ti­[CO_4_] can be formed by the reactions (TiO_2_ + CO_2_ = Ti­[CO_4_] and 2TiO_2_ + CO_2_ = Ti_2_O_3_[CO_3_]) of TiO_2_ with CO_2_. In most experiments we used synthetic TiO_2_ samples as starting material. In the case of natural samples, we obtained the same results. While a more detailed analysis of the reaction would be desirable, it should be noted that the reactions take place with molten CO_2_. There is currently no *p*, *V, T*-data available for CO_2_ at the conditions employed here, and hence properties such as reaction volumes at high pressures and high temperatures cannot be reliably obtained.

Currently we can only present a few constraints based on our experiments regarding the stability field of both Ti-carbonates. During brief heating at pressures ranging from 25 to 35 GPa, Ti-carbonates were not readily formed. This might be the result of too low temperatures or these conditions are just outside the stability field of carbonate formation. Therefore, the determination of the stability field of Ti­[CO_4_] and Ti_2_O_3_[CO_3_] requires further theoretical and experimental studies.

## Conclusions

We provided a detailed study of the synthesis, crystal structures, Raman data and elastic properties of two new Ti-carbonates, Ti_2_O_3_[CO_3_] and Ti­[CO_4_]. Both structures contain Ti^4+^-cations and are the first two examples of anhydrous, chemically simple carbonates incorporating a tetravalent transition metal cation. We have solved both crystal structures and established their characteristic Raman signatures.

While Ti_2_O_3_[CO_3_] contains isolated trigonal [CO_3_]^2–^, the Ti­[CO_4_]-structure is characterized by the presence of isolated [CO_4_]^4–^-carbonate groups. Both structures contain 8-fold coordinated tetravalent Ti^4+^-cations. The bulk modulus of Ti­[CO_4_] is around *K*_0_ ≈ 200 GPa, while Ti_2_O_3_[CO_3_] has a bulk modulus in the range of *K*_0_ ≈ 131–162 GPa. However, further experimental studies with X-diffraction are required to determine the bulk modulus more precisely. The compact packing of edge-sharing and incompressible [TiO_8_] polyhedra with CO_4_-groups explains the large incompressibility of Ti­[CO_4_] compared to other carbonates. The tilting of [TiO_8_]-polyhedra against [CO_4_]- or [CO_3_]-groups governs the compression behavior, and explains the anisotropic compressibilities of the unit cells of Ti­[CO_4_] and Ti_2_O_3_[CO_3_].

Ti­[CO_4_]-*I*41/*amd* is isostructural to zircon, Zr­[SiO_4_], and the pressure-induced phase transition seems to be similar to the phase transition in Zr­[SiO_4_] transforming from *I*4_1_/*amd*to *I*4̅2*d* under compression. It would be desirable to better understand the volume change at the transition. However, as the predicted volume change is smaller than 1%, it will be challenging to detect the transition using X-diffraction, similar to what has been the case for Zr­[SiO_4_].^65,66^

Since the amount of carbon in the mantle and the incorporation of carbon in mantle minerals is still being discussed controversially^76^ the synthesis and characterization of anhydrous Ti-bearing carbonates constitutes a significant extension of the established crystal chemistry of carbonates with the potential to have a significant impact on our understanding of the deep carbon cycle. The simultaneous existence of CO_2_ and TiO_2_ in inclusions of diamonds offers direct evidence for the presence of TiO_2_ and CO_2_ in the mantle at conditions where the diamonds are forming.^37^ Hence, once the stability fields of the Ti-carbonates obtained here are better constrained, their role as carbon hosts in the deep carbon cycle should be reassessed.

## Supplementary Material



## Notes

Additional experimental details, the results of the single crystal structure solution, and DFT-based calculations, including cif-files (PDF)
