# Supplementary material for: Synthesis and Characterization of Ti4+ Containing Carbonates Ti[CO4] and Ti2O3[CO3]
Source: Inorg Chem. 2025 Oct 27;64(44):21893–902. doi: 10.1021/acs.inorgchem.5c03033 (PMC12674196; doi:10.1021/acs.inorgchem.5c03033)
Supplement: Supplementary file 1 [file ic5c03033_si_001.pdf]

*bayarjargal@kristall.uni-frankfurt.de

Supporting Information: Synthesis and characterization of Ti^{4+} containing carbonates $Ti[CO_4]$ and $Ti_2O_3[CO_3]$

Lkhamsuren Bayarjargal[*,a], Dominik Spahr[a], Victor Milman[b], Nico Giordano[c], Konstantin Glazyrin[c], Björn Winkler[a]

[a]*Institute of Geosciences, Goethe University Frankfurt, Altenhöferallee 1, 60438 Frankfurt, Germany*
[b]*Dassault Systèmes BIOVIA, 334 Cambridge Science Park, Cambridge CB4 0WN, United Kingdom*
[c]*Deutsches Elektronen-Synchrotron DESY, Notkestr. 85, 22607 Hamburg, Germany*

1. TiO_2-OII

1.1. Single crystal synchrotron X-ray diffraction

We measured 126 independent reflections of with an intensity of $I > 2\sigma(I)$ and refined 13 parameters. The resulting reflection to parameter ratio of 9.7 ($\approx$ 126 : 13) allows a reliable structure refinement. The wR-value after the refinement was 5.4%. The crystallographic data of TiO_2-OII have been deposited in the Cambridge Structural Database (CSD) under deposition numbers CCDC 2485339.

Table. S 1: Structural parameters of TiO_2-OII obtained from single crystal structure solution. Data collection was performed on quenched samples at 42(3) GPa at ambient temperature.

**Crystal data**	
Chemical formula	TiO_2-OII
Crystal system	Orthorhombic
Space group	*Pnma* (62)
Formula weight M_r	
Z	4
a (Å)	5.1097(7)
b (Å)	2.9612(13)
c (Å)	5.944(4)
α (°)	90
β (°)	90
γ (°)	90
V (Å^3)	89.94(7)
ρ (g cm^{-3})	5.8992
CCDC number	2485339
**Data collection**	
Wavelength (Å)	0.2901
2θ range (°)	6.28 to 33.60
measured reflections	257
independent reflections	126
reflections $I > 2\sigma(I)$	126
R_{int}	0.0181
**Refinement**	
$R[F^2 > 2\sigma(F^2)]$, $wR(F^2)$	0.0393, 0.0542
No. of reflections	126
No. of parameters	13
No. of restraints	0

Table. S 2: Atomic coordinates of TiO_2-OII at 42(3) GPa obtained by single crystal structure refinement.

**Atom**	x	y	z	U_{iso} (Å^2)
Ti1	0.24304(16)	0.25	0.11855(19)	0.0045(4)
O1	0.3586(7)	0.25	0.4289(8)	0.0035(6)
O2	0.0286(6)	0.75	0.3387(9)	0.0050(6)

**Atom**	U_{11}	U_{22}	U_{33}	U_{23}	U_{13}	U_{12}
Ti	0.0039(3)	0.0015(7)	0.0080(8)	0	-0.0010(4)	0

1.2. *Equation of state of high pressure phases of TiO_2-OII*

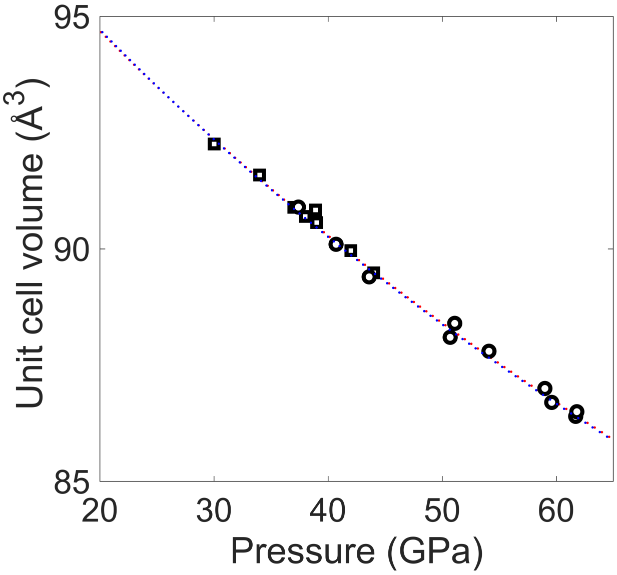

Figure S 1: Pressure-dependence of the unit-cell volume (circles) of TiO_2-OII phase. The dashed red line represents the second-order Birch-Murnaghan equation of state (using K_0 = 306 GPa, $V_0 = 100.36$ Å^3) and the dashed blue line represents the third-order Birch-Murnaghan equation of state with K_0 = 294 GPa, $V_0 = 100.56$ Å^3 and $K_p = 4.25$ based on the previous study [1]. Circles represent the result of the current study, while squares represent data from the previous study [1].

2. $Ti_2O_3[CO_3]$

2.1. *Single crystal synchrotron X-ray diffraction*

We collected single crystal X-ray diffraction data suitable for the crystal structure solution. Fig. S 2 (a) shows a part of an *unwarped* image of the raw-experimental data after processing of the ($h0l$) area in CrysAlis on the selected location with the lattice grid and reflections of $Ti_2O_3[CO_3]$. Fig. S 2 (b) and (c) show two projections of reciprocal space and the distribution of reflections.

We measured 311 independent reflections of with an intensity of $I > 2\sigma(I)$ and refined 24 parameters. The resulting reflection to parameter ratio of 13 ($\approx$ 311 : 24) allows a reliable structure refinement. The wR-value after the refinement was 5.4%. DFT calculations reproduced the monoclinic space group $P2/c$ of $Ti_2O_3[CO_3]$. Comparisons of the crystallographic parameters to DFT calculations can be found in Tab.S 3 and Tab.S 4 lists. The agreement between the experimental and DFT-calculated lattice parameters is convincing. The crystallographic data of $Ti_2O_3[CO_3]$ have been deposited in the Cambridge Structural Database (CSD) under deposition numbers CCDC 2416531.

Due to the metallic body of the DAC the access to the reciprocal space is very limited. Nevertheless, the displacement parameters of all Fe atoms could be refined anisotropically and no constraints or restraints had to been introduced. We refined the displacement parameters of all C and O atoms isotropically. In addition, the isotropic displacement parameters of the symmetrically independent carbon and oxygen atoms were constrained to be identical, in order to reduce the number of free parameters. A DAC typically shades more than 60 % of the reflections and hence the access to the reciprocal space in the DAC is limited and the number of reflections is moderate.

3. $Ti[CO_4]$

3.1. *Single crystal synchrotron X-ray diffraction of $Ti[CO_4]$*

We collected single crystal X-ray diffraction data suitable for the crystal structure solution. Fig. S 4 (a) shows a part of an *unwarped* image of the raw-experimental data after processing of the ($h0l$) area in CrysAlis on the selected location with the lattice grid and reflections of $Ti[CO_4]$ at 14.4(3.0) GPa. Fig. S 4 (b) and (c) show two projections of reciprocal space and the distribution of reflections.

We measured 180 independent reflections of with an intensity of $I > 2\sigma(I)$ and refined 8 parameters for $Ti[CO_4]$-$I\bar{4}2d$. 260 independent reflections of $Ti[CO_4]$-$I4_1/amd$ were refined with 8 parameters. The resulting reflection to parameter ratios 22 ($\approx$ 180 : 8) for $Ti[CO_4]$-$I\bar{4}2d$ and 37 ($\approx$ 260 : 7) for $Ti[CO_4]$-$I4_1/amd$ allow reliable structure refinements. The wR-values after the refinement were 4.2% and 9.8 % for the space groups $I\bar{4}2d$ and $I4_1/amd$,

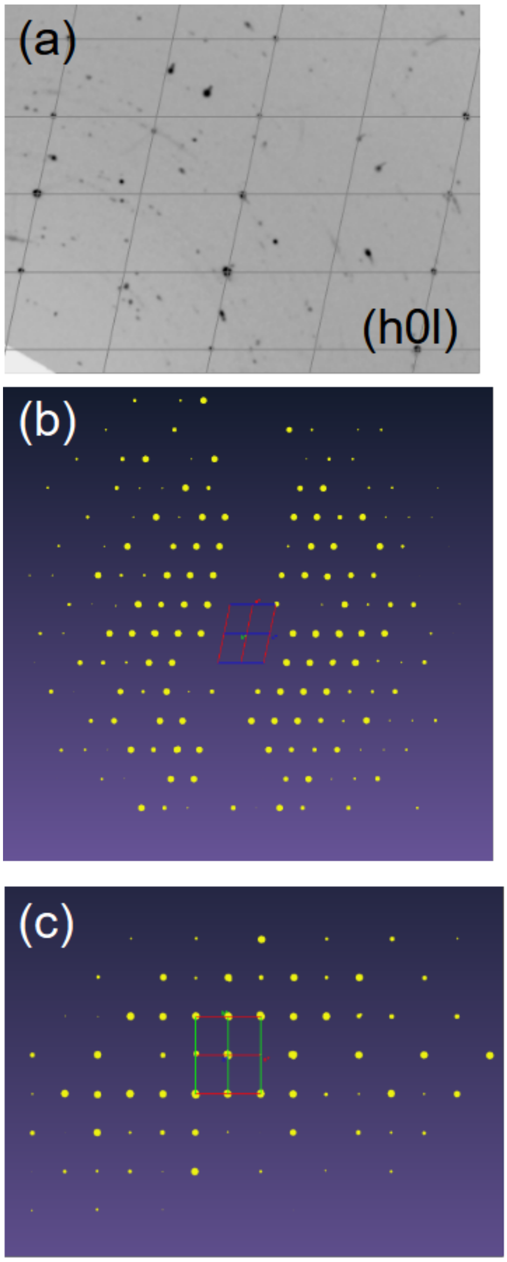

Figure S 2: $Ti_2O_3[CO_3]$: (a) *Unwarped* image of the raw-experimental data after data reduction. The ($h0l$) area and some indexed reflections are shown. (b) Schematic depiction of the reflections in reciprocal space using the Ewald-Explorer in CrysAlis after data reduction which were later used for the refinement. Projections of the reciprocal space are shown along b^*. (c) Schematic depiction of the reflections in a^* and c^* plane. The diameter of the reflections is proportional to their intensity.

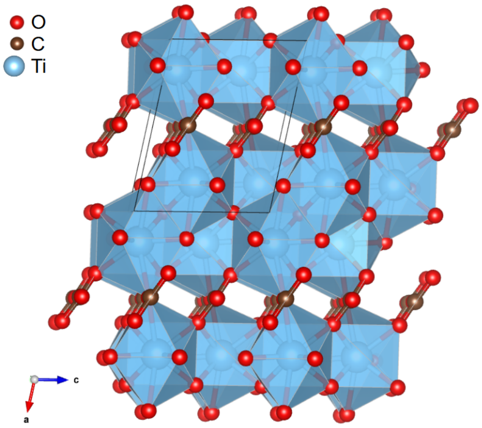

Figure S 3: The structure of $Ti_2O_3[CO_3]$ at 39(3) GPa, viewed along [010]. The structure contains isolated CO_3-groups and TiO_8-polyhedra. Each two layers of TiO_8-polyhedra are connected to each other with CO_3-groups.

Table. S 3: Structural parameters of $Ti_2O_3[CO_3]$ obtained from DFT calculations and single crystal structure solution. Data collection was performed on quenched samples at 39(3) GPa at ambient temperature.

	**Experiment**	**DFT**
Presssure (GPa)	**39(3)**	**40**
**Crystal data**		
Chemical formula	$Ti_2O_3[CO_3]$	
Crystal system	Monoclinic	
Space group	$P2/c$ (13)	
Formula weight M_r	203.76	
Z	2	
a (Å)	6.4859(10)	6.4614
b (Å)	4.213(3)	4.3257
c (Å)	4.990(2)	5.0041
α (°)	90	90
β (°)	101.71(2)	101.7846
γ (°)	90	90
V (Å^3)	133.51(11)	136.9169
ρ (g cm^{-3})	5.0686	4.9423
CCDC number	2416531	
**Data collection**		
Wavelength (Å)	0.2901	
2θ range (°)	5.24 to 34.24	
measured reflections	613	
independent reflections	382	
reflections $I > 2\sigma(I)$	311	
R_{int}	0.0119	
**Refinement**		
$R[F^2 > 2\sigma(F^2)]$, $wR(F^2)$	0.0393, 0.0542	
No. of reflections	311	
No. of parameters	24	
No. of restraints	0	
$\Delta\rho_{max}$, $\Delta\rho_{min}$ (e Å^{-3})	1.06, -0.94	

Table. S 4: Atomic coordinates of $Ti_2O_3[CO_3]$ at 39(3) GPa obtained by single crystal structure refinement.

**Atom**	x	y	z	U_{iso} (Å^2)
Ti1	0.82541(10)	0.7295(3)	0.90654(14)	0.0056(3)
O1	0.8449(4)	0.1157(13)	0.0658(6)	0.0054(5)
O2	0.6393(4)	0.6078(13)	0.1651(6)	0.0054(5)
O3	0.5	-0.0569(18)	0.75	0.0057(7)
C1	0.5	0.231(2)	0.75	0.0049(8)
O4	1	0.5804(19)	0.25	0.0070(7)

Table. S 5: Anisotropic displacement parameters Å^2 of $Ti_2O_3[CO_3]$ at 39(3) GPa obtained by single crystal structure refinement.

**Atom**	U_{11}	U_{22}	U_{33}	U_{23}	U_{13}	U_{12}
Ti	0.0059(3)	0.0057(9)	0.0054(3)	0.0001(3)	0.00135(14)	0.0000(4)

Table. S 6: Atomic coordinates of $Ti_2O_3[CO_3]$ at 40 GPa obtained by DFT calculations.

Atom	x	y	z
Ti1	0.82621	0.73513	0.90436
O1	0.84641	0.11223	0.06468
O2	0.63981	0.61101	0.16532
O3	0.5	-0.06184	0.75
C1	0.5	0.22985	0.75
O4	1	0.57477	0.25

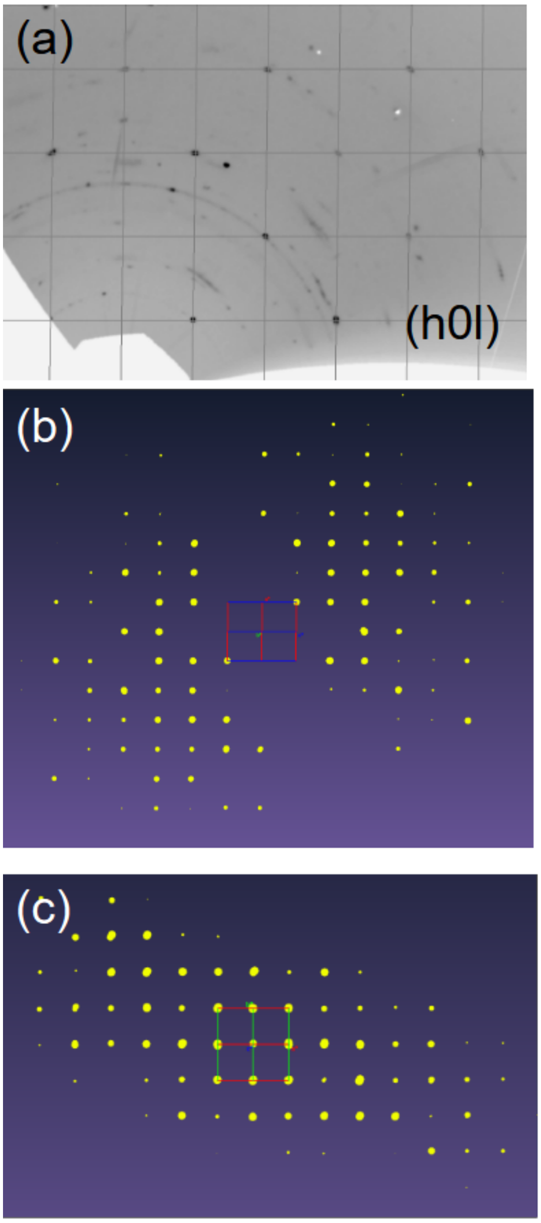

Figure S 4: Ti[CO_4] at 14.4(3.0) GPa: (a) *Unwarped* image of the raw-experimental data after data reduction. The ($h0l$) area and some indexed reflections are shown. Schematic depiction of the reflections in reciprocal space using the Ewald-Explorer in CrysAlis after data reduction which were later used for the refinement. Projections of the reciprocal space are shown along b^* (b) and c^* (c). The diameter of the reflections is proportional to their intensity.

respectively. DFT calculations reproduced the both crystal structures of Ti[CO_4] with the tetragonal space group. Comparisons of the crystallographic parameters to DFT calculations can be found in Tab.S 8 –7 lists.

The crystallographic data of Ti[CO_4] have been deposited in the Cambridge Structural Database (CSD) under deposition numbers CCDC 2416536 and 2416537.

Table. S 7: Structural parameters of Ti[CO_4] obtained from DFT calculations and single crystal structure solution. Data collection was performed on quenched samples at 39(3) GPa and 14.4(3.0) GPa at ambient temperatures.

Presssure (GPa)	**Experiment** **39(3)**	**DFT** **40**	**Experiment** **14.4(3.0)**	**DFT** **10**
**Crystal data**				
Chemical formula	Ti[CO_4]		Ti[CO_4]	
Crystal system	Tetragonal		Tetragonal	
Space group	$I\bar{4}2d$ (122)		$I4_1/amd$ (141)	
Formula weight M_r	123.89		123.89	
Z	4		4	
a (Å)	5.8124(9)	5.8589	6.021(4)	6.12940
b (Å)	5.8124(9)	5.8589	6.021(4)	6.12940
c (Å)	5.080(3)	5.1350	5.185(2)	5.21090
α (°)	90	90	90	90
β (°)	90	90	90	90
γ (°)	90	90	90	90
V (Å^3)	171.62(11)	176.2677	187.9(2)	195.7711
ρ (g cm^{-3})	4.7947		4.379	
CCDC number	2416536		2416537	
**Data collection**				
Wavelength (Å)	0.2901		0.2904	
2θ range (°)	4.34 to 33.96		4.24 to 24.46	
measured reflections	333		260	
independent reflections	180		76	
reflections $I > 2\sigma(I)$	180		63	
R_{int}	0.0067		0.0304	
**Refinement**				
$R[F^2 > 2\sigma(F^2)]$, $wR(F^2)$	0.0307, 0.0428		0.0379, 0.0982	
No. of reflections	180		260	
No. of parameters	8		7	
No. of restraints	0		0	
$\Delta\rho_{max}$, $\Delta\rho_{min}$ (e Å^{-3})	0.59, -0.59		0.851, -1.148	

Table. S 8: Atomic coordinates and atomic displacement parameters of Ti[CO_4]-$I\bar{4}2d$ obtained by single crystal structure refinement at 39(3) GPa and DFT calculations at 40 GPa.

39(3) GPa		Experiment					
**Atom**	x	y	z	U_{iso} (Å^2)	U_{11} (Å^2)	U_{22} (Å^2)	U_{33} (Å^2)
Ti	1	0.5	0.75	0.0043(2)	0.0060(2)	0.0060(2)	0.0008(6)
O	0.6778(3)	0.4716(3)	0.6694(5)	0.0061(3)			
C	0.5	0.5	0.5	0.0051(6)			

40 GPa		DFT	
Ti	1	0.5	0.75
O	0.6769	0.47033	0.6687
C	0.5	0.5	0.5

Table. S 9: Atomic coordinates and atomic displacement parameters of Ti[CO_4]-$I4_1/amd$ at 14.4(3.0) GPa obtained by single crystal structure refinement and DFT calculations.

14.4(3.0) GPa		Experiment					
**Atom**	x	y	z	U_{iso} (Å^2)	U_{11} (Å^2)	U_{22} (Å^2)	U_{33} (Å^2)
Ti	0.5	0.75	0.375000	0.0031(6)	0.0013(7)	0.0013(7)	0.0068(8)
O	0.5	0.4243(5)	0.2959(5)	0.0046(7)			
C	0.5	0.25	0.125	0.0034(15)			
10 GPa		DFT					
Ti	0	1	1				
O	0	0.82706	0.32899				
C	0	1	0.5				

4. Elastic Stiffness Constants of $Ti_2O_3[CO_3]$ and Ti[CO_4]

Table. S 10: Elastic Stiffness Constants C_{ij} (GPa) of Ti[CO_4] at ambient pressure.

Ti[CO_4]-$I\bar{4}2d$					
281.81520	32.44270	153.49520	0.00000	0.00000	0.00000
32.44270	281.81520	153.49520	0.00000	0.00000	0.00000
153.49520	153.49520	630.98325	0.00000	0.00000	0.00000
0.00000	0.00000	0.00000	135.47615	0.00000	0.00000
0.00000	0.00000	0.00000	0.00000	135.47615	0.00000
0.00000	0.00000	0.00000	0.00000	0.00000	60.53105

Ti[CO_4]-$I4_1/amd$					
281.82490	31.36024	152.87388	0.00000	0.00000	0.00000
31.36024	281.82490	152.87388	0.00000	0.00000	0.00000
152.87388	152.87388	631.93276	0.00000	0.00000	0.00000
0.00000	0.00000	0.00000	137.39410	0.00000	0.00000
0.00000	0.00000	0.00000	0.00000	137.39410	0.00000
0.00000	0.00000	0.00000	0.00000	0.00000	62.99912

5. Compressibility of $Ti_2O_3[CO_3]$ and $Ti[CO_4]$

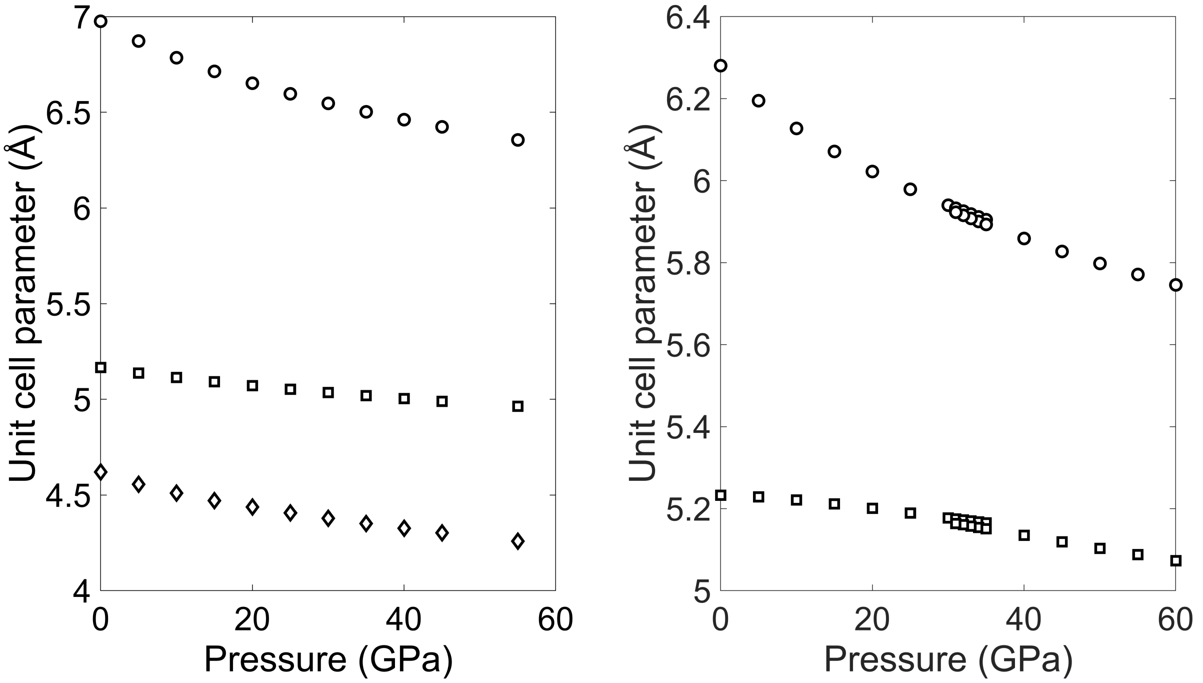

(a) (b)

Figure S 5: (a) Pressure dependence of the unit-cell parameters of $Ti_2O_3[CO_3]$ (a) and $Ti[CO_4]$ (b). Circles, diamonds and squares refer to the a, b and c unit-cell parameters, respectively.

The degree of the distortion of a coordination polyhedron can be described using an index D, with $D = \frac{1}{n}\sum_{i=1}^{n}\frac{|l_i - l_{av}|}{l_{av}}$, where l_i is the distance from the central atom to the ith coordinating atom and l_{av} is the average bond length[2]. These $[CO_4]$ tetrahedra of $Ti[CO_4]$ are not distorted (D = 0.0) in the whole pressure range and the C-O bond lengths decreases slightly by ∼4 % between 60 and 0 GPa (Fig.6). The $[TiO_8]$ polyhedra of $Ti[CO_4]$ are regular shaped, while the $[TiO_8]$ polyhedra of $Ti_2O_3[CO_3]$ form distorted polyhedra which is stretched out in two directions. The distortion of the $[TiO_8]$ polyhedra of $Ti[CO_4]$ at 65 GPa is D = 0.020 and decreases slightly to 0.011 up to 30 GPa. During the phase transition between 30 GPa and 15 GPa D drops to zero and increase back below 15 GPa to D = 0.022. In contrast, the distortion index the $[TiO_8]$ polyhedra of $Ti_2O_3[CO_3]$ is D = 0.053 at 65 GPa and increases up to 0.095 at 0 GPa.

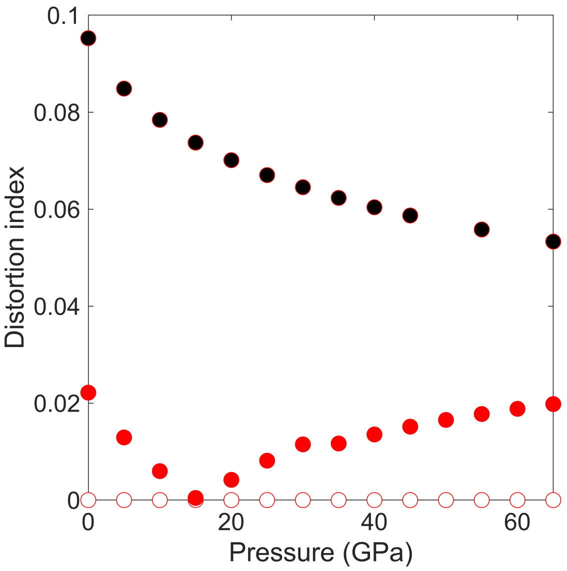

(a)

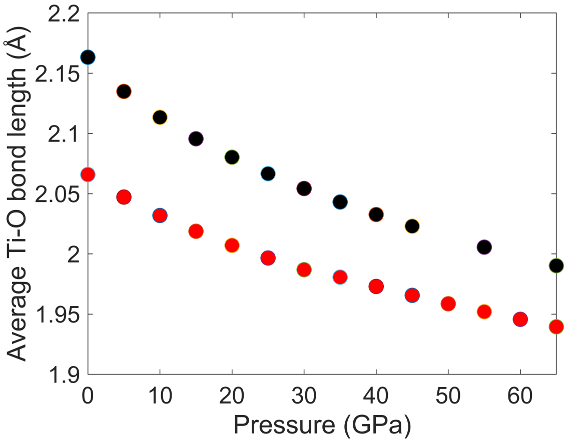

(b)

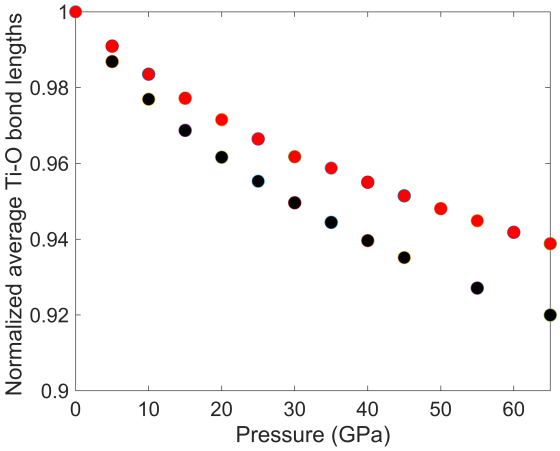

(c)

Figure S 6: Red circles represent Ti[CO_4], while black circles refer to Ti_2O_3[CO_3]. (a) Pressure-dependence of the distortion index D of the [TiO_8] polyhedra in Ti_2O_3[CO_3] and Ti[CO_4]. Open red circles mark the distortion index D of the [CO_4]-groups in Ti[CO_4]. (b) Pressure-dependence of the average Ti-O bond lengths in the [TiO_8] polyhedra. (c) Pressure-dependence of the normalized average Ti-O bond lengths in the [TiO_8] polyhedra.

6. EDX measurements of starting materials

We used a Phenom World ProX desktop SEM for imaging and energy dispersive X-ray spectroscopy (EDX) measurements, which allow a semi-quantitative chemical characterization of the starting materials (a synthetic TiO_2 powder with a purity of 99.5% from Alfa Aesar and natural single rutile crystals from Großes Zirknitztal, Austria). The samples were measured under low vacuum conditions to reduce charging effects on the sample with an acceleration voltage of 15 kV. Si powder was measured as a reference to detect possible errors or contamination of the measurement device. The Si measurement has a small impurity peak at 0.28 keV (C Kα), which can also be seen in the EDX measurements of TiO_2. The results of the EDX analysis are in agreement with the expected chemical composition for synthetic titanium oxide and rutile crystals (synthetic vs. natural EDX in wt.%, Ti: 56/57(4) and O: 44/43(5)) (See Fig.S 7). We did not detect any other elements within the sensitivity range of the device.

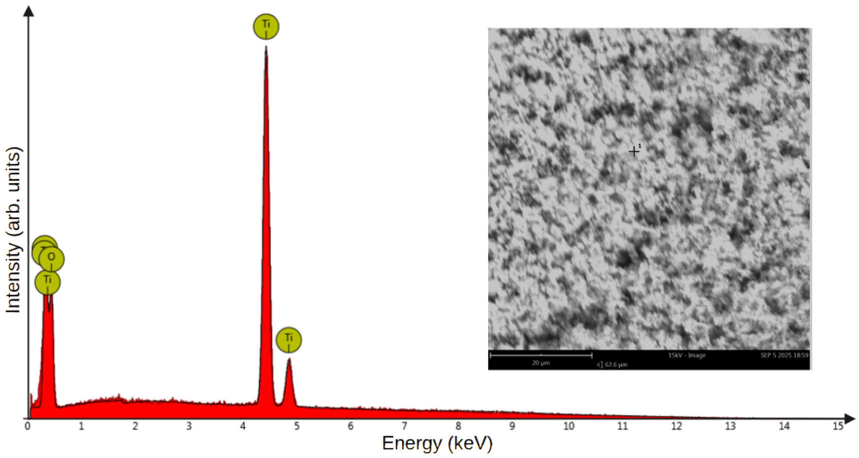

(a)

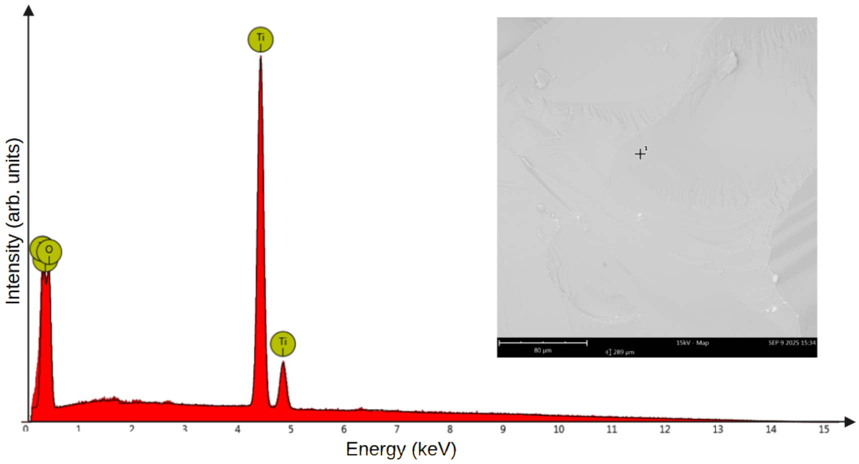

(b)

Figure S 7: SEM images and EDX spectra of the synthetic TiO_2 powder (a) and the natural single rutile crystal (b)

7. DFT calculations

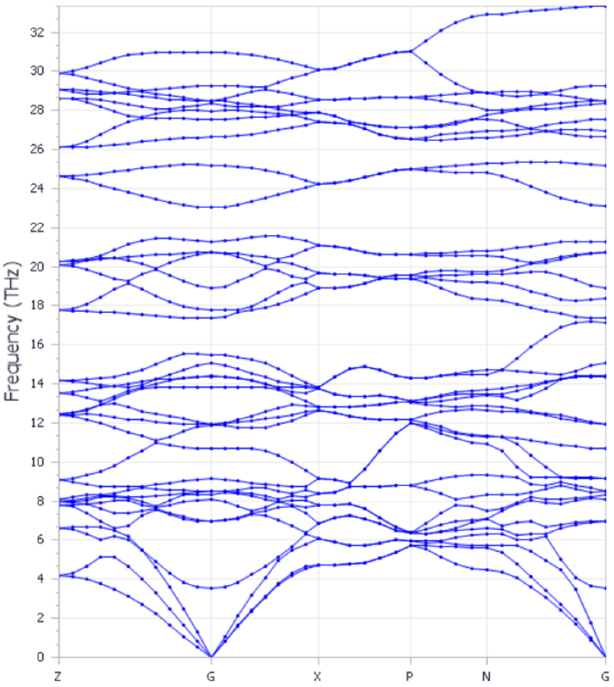

(a)

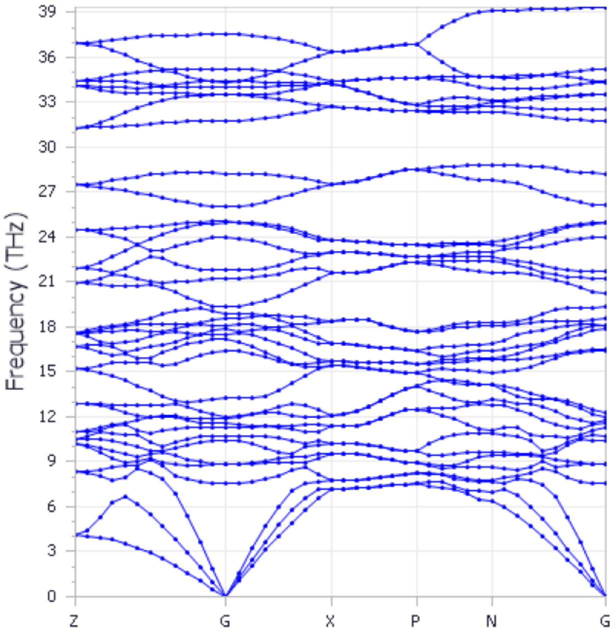

(b)

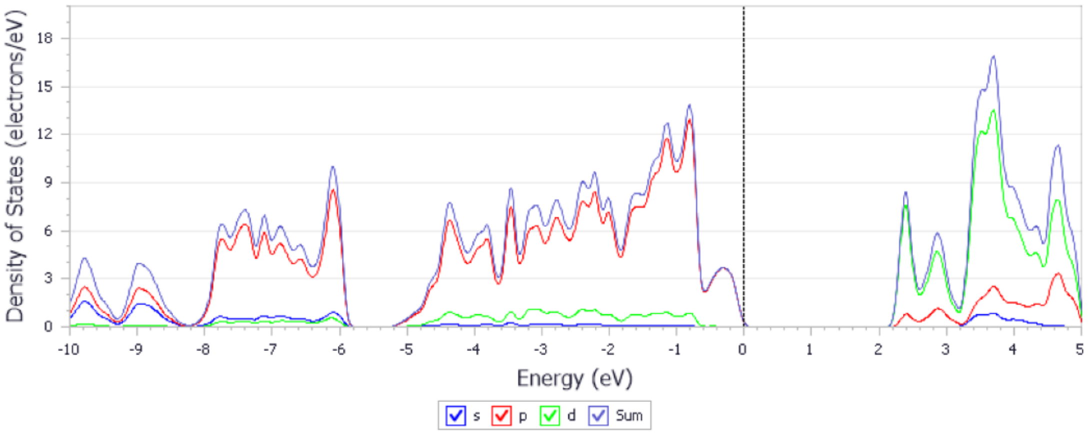

(c)

Figure S 8: (a) Phonon dispersion curves for Ti[CO_4]-$I4_1/amd$ at 0 GPa. (b) Phonon dispersion curves for Ti[CO_4]-$I\bar{4}2d$ at 60 GPa (c) Partial density of states of Ti[CO_4]-$I4_1/amd$, with s,p,d - contributions at 0 GPa.

References

[1] D. Nishio-Hamane, A. Shimizu, R. Nakahira, K. Niwa, A. Sano-Furukawa, T. Okada, T. Yagi, T. Kikegawa, The stability and equation of state for the cotunnite phase of TiO_2 up to 70 GPa, Phys. Chem. Miner. 37 (2010) 129–136.

[2] W. Baur, The geometry of polyhedral distortions. Predictive relationships for the phosphate group, Acta Crystallogr. B. 30 (5) (1974) 1195–1215.
